# Phytotoxic Activity of *Ocimum tenuiflorum* Extracts on Germination and Seedling Growth of Different Plant Species

**DOI:** 10.1155/2014/676242

**Published:** 2014-06-17

**Authors:** A. K. M. Mominul Islam, Hisashi Kato-Noguchi

**Affiliations:** Faculty of Agriculture, Kagawa University, 2393 Ikenobe, Miki, Kita, Kagawa 761-0795, Japan

## Abstract

Phytotoxic activity of *Ocimum tenuiflorum* (Lamiaceae) plant extracts was investigated against the germination and seedling growth of cress (*Lepidium sativum*), lettuce (*Lactuca sativa*), alfalfa (*Medicago sativa*), Italian ryegrass (*Lolium multiflorum*), barnyard grass (*Echinochloa crus-galli*), and timothy (*Phleum pratense*) at four different concentrations. The plant extracts at concentrations greater than 30 mg dry weight equivalent extract mL^−1^ reduced significantly the total germination percent (GP), germination index (GI), germination energy (GE), speed of emergence (SE), seedling vigour index (SVI), and coefficient of the rate of germination (CRG) of all test species except barnyard grass and GP of lettuce. In contrast, time required for 50% germination (*T*_50_) and mean germination time (MGT) were increased at the same or higher than this concentration. The increasing trend of *T*_50_ and MGT and the decreasing trend of other indices indicated a significant inhibition or delay of germination of the test species by *O. tenuiflorum* plant extracts and vice versa. In addition, the shoot and root growth of all test species were significantly inhibited by the extracts at concentrations greater than 10 mg dry weight equivalent extract mL^−1^. The *I*_50_ values for shoot and root growth were ranged from 26 to 104 mg dry weight equivalent extract mL^−1^. Seedling growth was more sensitive to the extracts compared to seed germination. Results of this study suggest that *O. tenuiflorum* plant extracts have phytotoxic properties and thus contain phytotoxic substances. Isolation and characterization of those substances from this plant may act as a tool for new natural, biodegradable herbicide development to control weeds.

## 1. Introduction

Overuse of synthetic herbicides to control weeds lead to an increased risk of herbicide resistant weed biotypes [1] and harsh environmental pollutions [2–4]. Alternative weed management strategies that are ecofriendly and cost-effective are therefore a time demanding issue throughout the world. In this backdrop, phytotoxic plants might help in resolving the problems created by synthetic herbicides as they possess growth retarding substances. Recently, there has been an increasing interest shown by the researchers on phytotoxic medicinal plants [5–8]. The increasing interest on medicinal plants could be due to either (i) the easier screening process of phytotoxic plants from medicinal plants [6] or (ii) the possibility to have more bioactive compounds in medicinal plants than other plants. These phytotoxic plants could be used in several ways to control weeds, for example, (i) sowing/transplanting them as relay or cover crops with main crops, (ii) direct application of their crude extracts as bioherbicides, or (iii) isolation and characterization of their active substances and using them as a tool for new natural and biodegradable herbicides development.

*Ocimum tenuiflorum *L. syn.* O. sanctum *belonging to Lamiaceae family is a widely distributed perennial shrub throughout the tropical and subtropical Asia. Due to its multitude medicinal properties such as antidiabetic, antioxidant, antimicrobial, antinociceptive, antifertility, anti-inflammatory, anticancer, anthelmintic, and cardioprotective [9], the plant is designated as “Holy Basil” in India. It is grown in the courtyards and in front of temples by the Hindus for religious and medicinal purposes, besides being cultivated for essential oil production. The essential oil of this plant has either phenolic constituents, for example, eugenol, thymol, or sesquiterpene alcohols as single major oil constituents, or terpene compounds as minor constituents [10, 11]. Besides pharmacological properties, very few are known about the phytotoxic activity of* O. tenuiflorum*. Therefore, current research was undertaken to investigate and identify the phytotoxic properties of the aqueous methanol extract of* O. tenuiflorum* on germination and early seedling growth of six test plant species under control laboratory conditions.

## 2. Materials and Methods

### 2.1. Plant Materials

The whole plants (leaves, stems, and roots) of* O. tenuiflorum* were collected from Bangladesh in 2012. The plants were then washed with tap water to remove the soil and other debris, dried under sun, and kept at 2°C until extraction. Three dicotyledonous: cress (*Lepidium sativum* L.), lettuce (*Lactuca sativa* L.), and alfalfa (*Medicago sativa* L.), and three monocotyledonous: Italian ryegrass (*Lolium multiflorum* Lam.), barnyard grass (*Echinochloa crus-galli* L.), and timothy (*Phleum pratense *L.), were selected as test plant species. Those species were chosen on the basis of their (i) growth patterns, (ii) sensitivity to allelopathic extracts, and (iii) weedy characteristics.

### 2.2. Extraction Procedure

The plants of* O. tenuiflorum* (30 g) were cut into small pieces and extracted with 300 mL of 70% (v/v) aqueous methanol for 2 days. After filtration using one layer of filter paper (number 2; Advantec Toyo Roshi Kaisha, Ltd., Tokyo, Japan), the residue was extracted again with the same volume of methanol for 1 day and filtered. Two filtrates were mixed together and then evaporated with a rotary evaporator at 40°C.

### 2.3. Germination Bioassay

A portion of the extract was diluted into small volume of methanol to prepare four assay concentrations 3, 10, 30, and 100 mg dry weight equivalent extract mL^−1^ and then was added to a sheet of filter paper (number 2) in 28 mm Petri dishes. The methanol was evaporated in a draft chamber followed by adding 0.6 mL of 0.05% (v/v) aqueous solution of polyoxyethylene sorbitan monolaurate (Tween 20: a nontoxic surfactant for germination and growth of all test plants). Ten seeds of cress, lettuce, alfalfa, Italian ryegrass, barnyard grass, or timothy were placed on the filter paper in Petri dishes. Control Petri dishes were also maintained in each experiment using only Tween 20, that is, without plant extracts. The Petri dishes were then incubated in dark at 25°C. Germination was measured at every 0.5-day interval up to 4 days (the time when no further seeds germinated) and was considered when the radical emerge by rupturing the seed coat as per Islam and Kato-Noguchi [12].

Eight germination indices, that is, germination percentage (GP), germination index (GI), germination energy (GE), speed of emergence (SE), time required for 50% germination (*T*_50_), mean germination time (MGT), seedling vigour index (SVI), and coefficient of the rate of germination (CRG) were calculated from the same data by using the equations described in Table 1. GP index indicated the total germination percent of a seed lot after certain period of time when germination became constant. As it is measured by total germination relative to total number of seeds set for germination, GP cannot explain the delayed germination. In contrast, GI is a measure of both percentage and speed of germination and assigns maximum arithmetic weight to seeds that germinate during first count and less weight to those that germinate later. The higher the GI, GE, SE, SVI, and CRG values compared to control, the lower the inhibition, and vice versa. But the meaning is reversed for *T*_50_ and MGT.

### 2.4. Growth Bioassay

The Petri dishes and the extracts were prepared as described above. Ten seeds of cress, lettuce, or alfalfa or ten pregerminated seeds of Italian ryegrass, barnyard grass, or timothy (germinated in the darkness at 25°C for 1–3 days after overnight soaking) were placed on the filter paper in Petri dishes. The shoot and root lengths of each seedling were measured after incubation in dark condition for 2 days at 25°C. Control Petri dishes were also maintained as germination bioassay.

### 2.5. Statistical Analysis

The bioassay experiments were conducted as completely randomized design (CRD) with three replications. The experiments were repeated twice to avoid any experimental error. The data generated in each experiment were analyzed using statistical package SAS, version 9.01 (SAS Institute Inc., Cary, NC, USA). Treatments means were compared using Tukey's test at 5% level of probability [21]. The concentration required for 50% growth inhibition, that is, *I*_50_ of the test species in the assays, was calculated from the regression equation of the concentration response curves by GraphPad Prism 6.0 (GraphPad Software, Inc., La Jolla, California, USA).

## 3. Results

### 3.1. Effect of* O. tenuiflorum* Plant Extract on Germination

**Figure 1:**
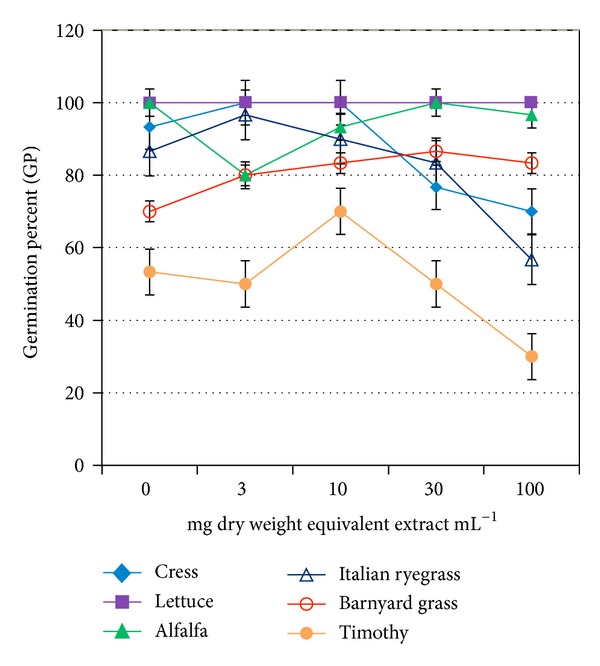
Effect of aqueous methanol extracts of* O. tenuiflorum* on GP of different plant species at four different concentrations. Concentrations of tested samples corresponded to the extract obtained from 3, 10, 30, and 100 mg dry weight of* O. tenuiflorum*. Vertical bars represent standard error deviations. Means ± SE from three independent experiments with 10 seedlings for each determination are shown.

**Figure 2:**
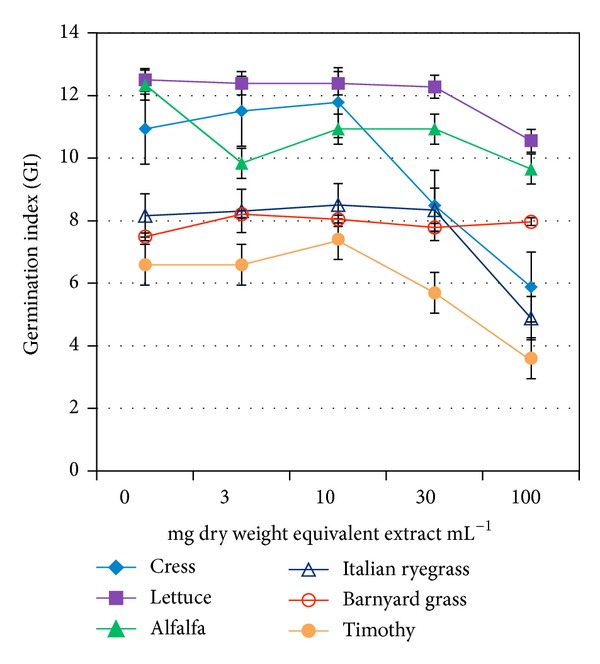
Effect of aqueous methanol extracts of* O. tenuiflorum* on GI of different plant species at four different concentrations. Other details are the same as Figure 1.

**Figure 3:**
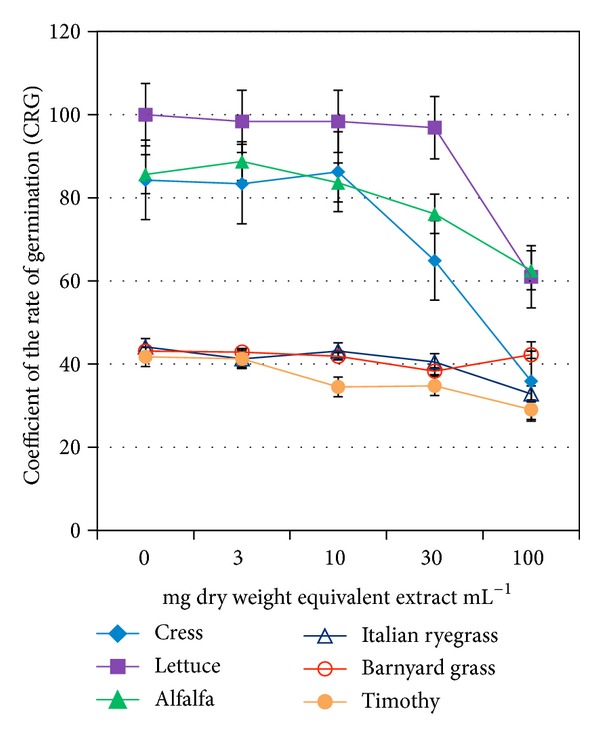
Effect of aqueous methanol extracts of* O. tenuiflorum* on CRG of different plant species at four different concentrations. Other details are the same as Figure 1.

**Figure 4:**
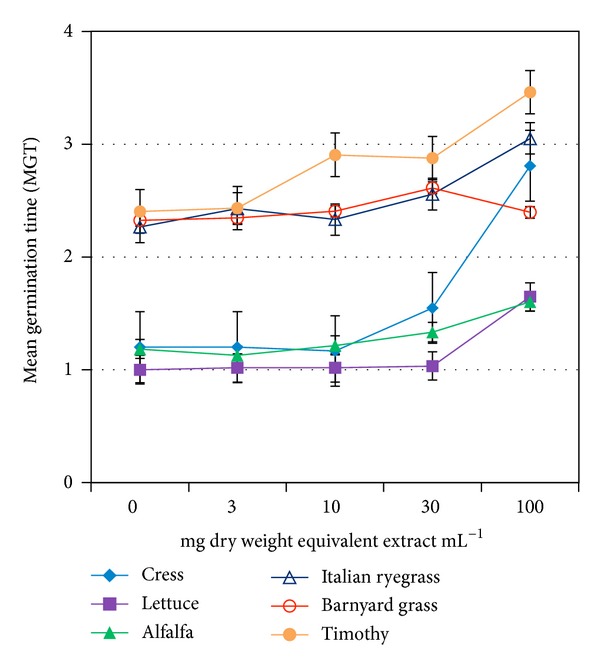
Effect of aqueous methanol extracts of* O. tenuiflorum* on MGT of different plant species at four different concentrations. Other details are the same as Figure 1.

The data generated in this study shows that the aqueous methanol extract of* O. tenuiflorum* at any concentration has significant (*P* < 0.001) effects on all calculated germination indices except *T*_50_. The GP of all but lettuce showed a reduction trend at concentration greater than 30 mg dry weight equivalent extract mL^−1^ (Figure 1). On the other hand, GI, GE, SE, SVI, and CRG of all but barnyard grass were decreased, whereas *T*_50_ and MGT were increased at the same or higher than that concentration (Tables 2(a) and 2(b) and Figures 2, 3, and 4). The increasing trend of *T*_50_ and MGT compared to the decreasing trend of GP, GI, GE, SE, SVI, and CRG indicated a significant inhibition or delay of germination of the test species caused by* O. tenuiflorum* plant extracts, and vice versa. However, the inhibitory activity on the germination was test plant species and concentration dependent.

### 3.2. Effect of* O. tenuiflorum* Plant Extract on Seedling Growth

**Figure 5:**
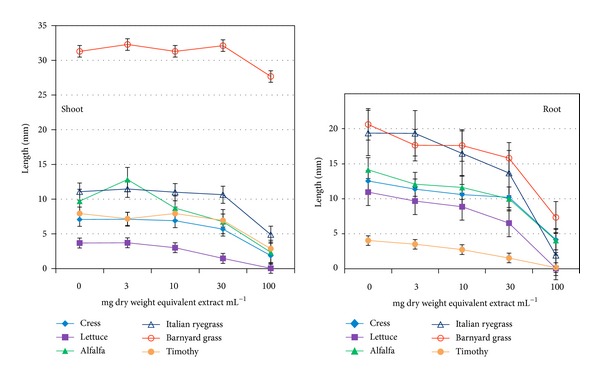
Effect of aqueous methanol extracts of* O. tenuiflorum* on shoot and root growth of different plant species at four different concentrations. Other details are the same as Figure 1.

Similar to germination,* O. tenuiflorum* plant extracts have significant effect (*P* < 0.001) on the seedling growth of cress, lettuce, alfalfa, Italian ryegrass, barnyard grass, and timothy. The aqueous methanol extracts of this plant showed inhibitory activity on the shoot and root growth of all the test species at concentrations greater than 10 mg dry weight equivalent extract mL^−1^ (Figure 5). The sensitivity of the seedling growth to the extracts was higher than the germination of the test species. In addition, the root growth was more sensitive than shoot; and the inhibitory activity of the extracts to the seedling growth was concentration and test plant species dependent (Figure 5). At 100 mg dry weight equivalent extract mL^−1^, the shoot growth of cress, lettuce, alfalfa, Italian ryegrass, barnyard grass, and timothy was inhibited by 24, 1, 25, 56, 91, and 46%, whereas that of the root growth was 47, 0, 29, 11, 37, and 10% of control, respectively. The *I*_50_ values for the shoot and root growth of the test species were ranged from 26 to 104 and 30 to 99 mg dry weight equivalent extract mL^−1^ (Table 3).

## 4. Discussion

Total germination percent (GP) is a commonly used index to measure the effects of phytotoxic substances [22, 23]. It is the maximum percentage of germination that mainly depends on final measurements. However, this index cannot interpret the possible delayed germination caused by phytotoxic plant extracts or substances. Therefore, GP is considered to be suitable for ecological studies rather than physiological process like germination [20, 22]. A number of indices over GP have been proposed by many researchers to study the inhibitory activity of phytotoxic substances on germination process [19, 24]. To investigate the actual inhibition (either direct inhibition or delayed effect) of* O. tenuiflorum *plant extracts on germination, we analysed few important germination indices: GI, SE, GE, SVI, CRG, *T*_50,_ and MGT together with GP. We observed a significant reduction of GI, SE, GE, SVI, and CRG and a promotion on *T*_50_ and MGT compared to control of all test species except barnyard grass. These results indicate the inhibitory potential of* O. tenuiflorum* plant extracts. The delay or inhibition of germination caused by phytotoxic plant extracts or substances was also reported by Anjum and Bajwa [24] and Hussain et al. [25].

Although germination bioassay is the most widely used method to inspect the phytotoxic activity [26, 27], early seedling growth is reported to be most sensitive parameter to test the phytotoxicity [28–30]. Hence, we have conducted the growth bioassay using the same test species to confirm the phytotoxic properties of* O. tenuiflorum* plant extracts. The bioassay results showed a significant reduction of shoot and root growth of all test species at 30 mg dry weight equivalent extract mL^−1^ or higher than this concentration. However, the sensitivity to the plant extracts was varied among the test species. The higher sensitivity of early seedling growth to phytotoxic plant extracts than to germination could be due to (i) the presence of seed coat which acts as a barrier in between the embryo and its surrounding environment [31], (ii) the selective permeability of seed coats [32] which may protect the inhibitory activity of phytotoxic extract/substances if they cannot pass through seed coats, (iii) the parameter that was used to measure germination (the protrusion of the root through the seed coat which does not necessarily mean growth by cell division), and so forth [33]. On the other hand, since roots are the first target tissue to confront with the phytotoxic substances, therefore inhibitory effects are more visible on roots rather than on shoots.

In summary, the aqueous methanol extract of* O. tenuiflorum* inhibited the seed germination of all but barnyard grass and the seedling growth of all test species. The inhibitory activities were concentration and test plant species dependent. These results indicated that* O. tenuiflorum* plant extracts have phytotoxic properties and thus contain phytotoxic substances. The concentration dependent inhibitory activities of allelopathic plant extracts on germination and seedling growth were also reported by Bogatek et al. [34] and Soltys et al. [35]. Therefore, the plant could be served as an important candidate for isolation and identification of allelopathic substances, which may promote the development of new natural herbicides. Besides this, the plant extracts or their residues could be directly used as bioherbicides. As the water extracts of this plant have growth stimulatory activity on* Brassica rapa* rather than inhibitory activity [36], farmers will get dual benefits from the plant residues such as bioherbicide for weeds and growth regulator for crops, when the residues are applied on their crop fields.

## 5. Conclusions

Weed management is one of the most challenging tasks in crop production. Overuse of synthetic herbicides causes severe environmental pollution besides being developed herbicide resistant weed biotypes. Plant product based natural herbicides could serve as an alternative to synthetic herbicides that are biodegradable and environment friendly. In this regard,* O. tenuiflorum *acts a promising role. Isolation and characterization of phytotoxic substances from* O. tenuiflorum* may promote the development of plant product based natural herbicides.

## Figures and Tables

**Table 1:** The equations used to calculate different germination indices.

Germination parameters	Equations	References
Germination percent (GP)	$\text{GP}=\left[\frac{\text{Number of germinated seeds at final count}}{\text{Total number of seeds sets for bioassay}}\right]\times 100$	Global method

Germination index (GI)	$\text{GI}=\sum \frac{G_{T}}{T_{t}}$ Or $\left[\frac{\text{Number of germinated seeds}}{\text{Days of first count}}\right]+\cdots +\left[\frac{\text{Number of germinated seeds}}{\text{Days of last or final count}}\right]$	AOSA [13]

Time required for 50% germination (*T*_50_)	$T_{50}=t_{i}+\frac{\left[\left\{(N/2)-n_{i}\right\}\left(t_{i}-t_{j}\right)\right]}{\left(n_{i}-n_{j}\right)}$ , where *N* is the final number of germination and *n*_*i*_, *n*_*j*_ cumulative numbers of seeds germinated by adjacent counts at times *t*_*i*_ and *t*_*j*_ when *n*_*i*_ < *N*/2 < *n*_*j*_	Coolbear et al. [14] modified by Farooq et al. [15]

Mean germination time (MGT)	$\text{MGT}=\left[\frac{\sum T_{i}/N_{i}}{\sum N_{i}}\right]$ ; here, *N*_*i*_ = number of newly germinated seeds at time *T*_*i*_	Ellis and Roberts [16]

Seedling vigour index (SVI)	$\text{SVI}=\left(\frac{\text{Seedling length}\left(\text{mm}\right)\;\times \text{ Germination percent}}{100}\right)$	Islam et al. [17]

Speed of emergence (SE)	$\text{SE}=\left(\frac{\text{Number of germinated seeds at the starting day of germiantion}}{\text{Number of germinated seeds at the final days of measurement}}\right)\times 100$	Modified from Islam et al. [17]

Germination energy (GE)	$\text{GE}=\left(\frac{\text{Percentage of germinated seeds at the starting day of germiantion}}{\text{Total number of seeds sets for bioassay}}\right)\times 100$	Modified from Ruan et al. [18]

Coefficient of the rate of germination (CRG)	$\text{CRG}=\left[\frac{\left(N_{1}+N_{2}+N_{3}+\cdots +N_{n}\right)}{\left(N_{1}\times T_{1}\right)+\left(N_{2}\times T_{2}\right)+\left(N_{3}\times T_{3}\right)+\cdots +\left(N_{n}\times T_{n}\right)}\right]\times 100$,	Bewley and Black [19], Chiapusio et al. [20]
	where *N*_1_ = number of germinated seeds on time *T*_1_ , *N*_2_ = number of germinated seeds on time *T*_2_ , *N*_*n*_ = number of germinated seeds on time *T*_*n*_	

**(a):** 

Test species	Time required for 50% germination (*T*_50_)					Speed of emergence (SE)				
	Control	3	10	30	100	Control	3	10	30	100
	mg dry weight equivalent extract mL^−1^					mg dry weight equivalent extract mL^−1^				
Cress	−2.3	−1.0	−2.7	1.8	2.4	74.8	66.7	76.7	31.9	4.2
Lettuce	0.0	−3.7	−3.7	−7.3	2.1	100.0	96.7	96.7	93.3	20.0
Alfalfa	−2.3	−2.0	−0.7	0.5	2.2	86.7	74.7	80.0	53.3	16.7
Italian ryegrass	1.3	2.0	1.9	2.1	2.7	15.28	13.70	10.00	14.44	0.00
Barnyard grass	2.3	2.3	2.3	2.4	1.6	14.48	25.26	19.91	18.33	26.98
Timothy	1.6	1.6	2.5	2.5	3.1	36.11	26.67	4.76	6.67	0.00
Statistical analysis										
*F*-value	∗∗	NS	NS	∗	NS	∗∗∗	∗∗∗	∗∗∗	∗∗∗	∗
CV (%)	1493.11	−2079.32	−5526.66	827.16	31.63	17.60	28.72	21.51	32.37	78.01
*R* ^2^	0.75	0.59	0.62	0.73	0.50	0.96	0.89	0.96	0.92	0.76
MSD	4.00	7.86	7.8367	8.21	2.12	27.23	41.23	29.27	33.34	25.00

**(b):** 

Test species	Seedling vigour index (SVI)					Germination energy (GE)				
	Control	3	10	30	100	Control	3	10	30	100
	mg dry weight equivalent extract mL^−1^					mg dry weight equivalent extract mL^−1^				
Cress	40.2	46.2	50.8	31.0	7.8	7.0	6.7	7.7	2.3	0.3
Lettuce	37.2	36.4	33.8	34.4	28.0	10.0	9.7	9.7	9.3	2.0
Alfalfa	39.3	24.9	35.2	35.3	25.4	8.7	6.0	7.3	5.3	1.7
Italian ryegrass	24.3	28.2	22.0	24.8	3.9	1.33	1.33	1.00	1.33	0.00
Barnyard grass	18.9	24.3	24.6	22.7	23.1	1.00	2.00	1.67	1.67	2.33
Timothy	1.3	1.5	1.5	0.9	0.1	2.00	1.33	0.33	0.33	0.00
Statistical analysis										
*F*-value	∗∗∗	∗∗∗	∗∗∗	∗∗	∗∗∗	∗∗∗	∗∗∗	∗∗∗	∗∗∗	∗
CV (%)	17.34	22.35	19.02	32.68	39.75	16.73	22.95	13.52	26.76	80.51
*R* ^2^	0.94	0.91	0.94	0.82	0.87	0.97	0.94	0.99	0.96	0.75
MSD	13.22	17.06	15.09	23.03	16.58	2.37	2.93	1.77	2.57	2.41

Note: Asterisks indicate a significant difference between control and treatment  *P < 0.05.  **P < 0.01 and  ***P < 0.001.

**Table 3:** *I*_50_ values of *O. tenuiflorum *plant extract for the shoot and root growth of different plant species.

Test species	*I*_50_ (mg dry weight equivalent extract mL^−1^)	
	Shoot	Root
Cress	86.81	99.05
Lettuce	26.39	35.53
Alfalfa	62.53	58.29
Italian ryegrass	Not converged	47.77
Barnyard grass	Not converged	75.69
Timothy	Not converged	30.34
